# Diseases Admitted under the Care of the Physicians of the Westminster Hospital, from Dec. 24, to March 25, 1801

**Published:** 1801-04

**Authors:** 


					Difeafes admitted under the Care of the Phyjicians of the Weflminjler
H'Jpital, from Dec. 24, to March 25, l8oi.
Continued Fevers 5Q
Intermittents   1
Pleurify   4
Small-pox   1
Amenorrhcea ?? 10
Anafarca    7
? Afcites    4
Afthenia   6
Afthma     2
.Catarrh     12
Cephalsea    6
Chlo?ofts '    3
Chorea ??? 1
Colic     * 4
Cough    2S
Diarrhoea   io
Dyfpcpfia   3
Dyl'pncea   z
Enterodynia   2
Galtrodynia    3
Hsematemefis   1
Haematuria   1
Haemoptyfis   I
Hasmorrhois   I
Hydrothorax   1
Hydrops ovarii I
Hyfteria.   4
Impetigo   9
Ifchuria   1
Itch      3
Jaundice
Lithiafis
Oblfipatio
3
3
r
Paralyfis   2
Phthifis   t.
Syncope 8c Palpitation z
Pleurodyne   i
Porl igra   I
Rheumatifm   ii)
Struma   13
T.ibes   z
Tinea      I
Worms   I
.The greateft part of the fevers were of the typhoid kind, as ufual at this
feafon of the year j but there were many of them that more refemhled the
autumnal bilious fevers, and in all of them there was an unufual tendency
to delirium, T. B?

				

